# Antiviral biflavonoids from *Radix Wikstroemiae *(*Liaogewanggen*)

**DOI:** 10.1186/1749-8546-5-23

**Published:** 2010-06-21

**Authors:** Weihuan Huang, Xiaoli Zhang, Yifei Wang, Wencai Ye, Vincent EC Ooi, Hau Yin Chung, Yaolan Li

**Affiliations:** 1 Department of Biology, The Chinese University of Hong Kong, Shatin, Hong Kong SAR, China; 2 Institute of Traditional Chinese Medicine and Natural Products, Jinan University, Guangzhou, 510632, China; 3 Guangdong Province Key Laboratory of Pharmacodynamic Constituents of Traditional Chinese Medicine and New Drug Research, Jinan University, Guangzhou, 510632, China; 4 Biomedicine Research and Development Center, Jinan University, Guangzhou, 510632, China

## Abstract

**Background:**

*Radix Wikstroemiae *is a common Chinese herbal medicine. The ethyl acetate fraction of the ethanolic extract of *W. indica *possesses potent *in vitro *antiviral activity against respiratory syncytial virus (RSV). This study aims to identify the antiviral components of the active fraction.

**Methods:**

The active fraction of the *Radix Wikstroemiae *extract was isolated with chromatographic methods such as silica gel, Sephadex LH-20 and semi-preparative high performance liquid chromatography (HPLC) columns. The structures of the isolated compounds were determined based on spectroscopic analyses. The *in vitro *antiviral activity of the compounds against RSV was tested with the cytopathic effect (CPE) reduction assay and the 3-(4,5-dimethylthiazol-2-yl)-2,5-diphenyltetrazolium bromide (MTT) method.

**Results:**

Four biflavonoids, namely neochamaejasmin B, genkwanol B, genkwanol C and stelleranol, were isolated and characterized. Genkwanol B, genkwanol C and stelleranol, which are stereo isomers of spirobiflavonoids, showed potent anti-RSV activity whereas neochamaejasmin B did not.

**Conclusion:**

Neochamaejasmin B, genkwanol B, genkwanol C and stelleranol were isolated from *Radix Wikstroemiae *and the complete absolute configurations of five chiral carbons in stelleranol were substantiated for the first time. Furthermore, the anti-RSV activity of genkwanol B, genkwanol C and stelleranol was reported for the first time.

## Background

The root of *Wikstroemia indica *(Linn.) C. A. Mey. (Thymelaeaceae) (*Radix Wikstroemiae*, *Liaogewanggen*) is a Chinese herbal medicine used to treat various inflammatory conditions [1]. *W. indica *tablet, an over-the-counter product made from the aqueous extract of *Radix Wikstroemiae*, is commercially available in China for the treatment of bronchitis, pneumonia, tonsillitis, parotitis and mastitis according to the Pharmacopoeia of China [2]. Moreover, *W. indica *have antifungal, anti-inflammatory, anti-cancer, antiviral and antimalarial effects [3-6]. Bioactive components of *W. indica *include (+)-nortrachelogenin, bis-5,5-nortrachelogenin, tricin, genkwanol A, kaempferol-3-*O*-beta-*D*-glucopyranoside, wikstrol A, wikstrol B, daphnodorin B, sikokianin B, sikokianin C, indicanone, lirioresinol B and daphnoretin [3-8]. In our previous study, the ethyl acetate fraction of the ethanolic extract of *Radix Wikstroemiae *possessed *in vitro *antiviral activity against RSV with IC_50 _value of < 3.9 μg/ml and selective index (SI) of > 64.1. Besides, daphnoretin (a dicoumarin compound) is one of the active components in the fraction, which mainly exerts its antiviral effects on the later phase of the viral replication cycle [9]. This article reports the isolation, structural characterization and *in vitro *antiviral tests of four biflavonoids, namely, (I) neochamaejasmin B, (II) genkwanol B, (III) genkwanol C and (IV) stelleranol from the ethyl acetate fraction of the ethanolic extract of *Radix Wikstroemiae*.

## Methods

### Plant material

*Radix Wikstroemiae *was purchased from a Chinese medicine pharmacy in Guangzhou, China. The authentication process was carried out by Zhengqiu Mai (Chinese Medicinal Material Company, Guangzhou, China) according to standard protocols [2]. A voucher specimen was deposited in the Institute of Traditional Chinese Medicine and Natural Products, College of Pharmacy, Jinan University (Guangzhou, China) with an accession number of 06111205.

### General experimental procedures

Optical rotations were determined on a Jasco P-1020 digital polarimeter (JASCO Corporation, Japan). The spectra of electrospray ionization-mass spectrometry (ESI-MS) were recorded on a Finnigan LCQ Advantage Max ion trap mass spectrometer (Thermo Finnigan, USA). The spectra of high resolution-electrospray ionization-mass spectrometry (HR-ESI-MS) were acquired with a Micromass Q-TOF mass spectrometer (Waters Corporation, USA). The spectra of nuclear magnetic resonance spectrometry (NMR) including proton magnetic resonance spectrometry (^1^H-NMR) and carbon magnetic resonance spectrometry (^13^C-NMR) were obtained on a Bruker spectrometer (Bruker Corporation, Switzerland) operating at 500 MHz for ^1^H-MNR and 125 MHz for ^13^C-NMR respectively. The isolation process was conducted on silica gel (200-300 meshes, Qingdao Marine Chemical, China), Sephadex LH-20 (25-100 μm, Fluka, Switzerland) and semi-preparative HPLC. Semi-preparative HPLC was performed on an Eclipse XDB-C_18 _column (9.4 mm ID × 25 cm) (Agilent Technologies, USA). Thin layer chromatography (TLC) was carried out on silica gel GF_254 _plates (0.2 mm thickness, 10 × 20 cm, Qingdao Marine Chemical, China) with FeCl_3_-EtOH reagent and ultraviolet (UV) illumination as chromogenic methods.

### Extraction and isolation

The dried and cut *Radix Wikstroemiae *(10.0 kg) was soaked in 95% ethanol at room temperature for three times (15 days each time). The ethanol solutions were then combined and concentrated *in vacuo *to yield a dark brown crude extract (1.0 kg). The ethanol extract was suspended in distilled water and partitioned with petroleum ether, ethyl acetate and *n*-butanol successively. After evaporation under reduced pressure, the petroleum ether fraction (5.0 g), ethyl acetate fraction (580.0 g) and butanol fraction (400.0 g) were obtained respectively.

The ethyl acetate fraction (550.0 g) was chromatographed on a silica gel column eluted with a solvent system of petroleum ether/ethyl acetate in gradient to obtain 28 subfractions based on the TLC analysis. The subfractions Fr-12 and Fr-23, which yielded positive reaction with FeCl_3_-EtOH reagent, were further isolated.

Fr-12 (12.6 g) was isolated with repeated silica gel columns eluted with gradient solvent systems of chloroform/acetone and chloroform/methanol, respectively, followed by Sephadex LH-20 column eluted with chloroform/methanol (3/7) to yield compound I (20.0 mg).

Fr-23 (12.6 g) was subjected to repeated silica gel columns eluted with gradient solvent system of chloroform/methanol, followed by semi-preparative HPLC on an Eclipse XDB-C_18 _column (Agilent Technologies, USA) with a gradient solvent system of 0.1% TFA (A) and methanol (B) to yield compounds II (40.7 mg), III (60.3 mg) and IV (20.7 mg).

### Identification

Compound I (neochamaejasmin B), an amorphous brown powder, was positive to FeCl_3_-EtOH reagent. [*α*]_D _= 200° (*c *0.1, MeOH). ESI-MS: *m/z *541 [M-H] ^- ^.^1^ H-NMR (500 MHz, CD_3_OD) *δ*: 7.17 (2H, d, *J *= 8.4 Hz, H-2''', H-6'''), 6.94 (2H, d, *J *= 8.4 Hz, H-2', H-6'), 6.81 (2H, d, *J *= 8.4 Hz, H-3''', H-5'''), 6.67 (2H, d, *J *= 8.4 Hz, H-3', H-5'), 6.00 (1H, d, *J *= 1.8 Hz, H-8''), 5.89 (1H, d, *J *= 1.8 Hz, H-8), 5.80 (1H, d, *J *= 1.8 Hz, H-6''), 5.78 (1H, d, *J *= 1.8 Hz, H-6), 5.57 (1H, d, *J *= 4.6 Hz, H-2), 5.16 (1H, d, *J *= 8.8 Hz, H-2''), 3.29 (1H, dd, *J *= 3.2, 8.8 Hz, H-3''), 3.16 (1H, brs, H-3). ^13^C-NMR (125 MHz, CD_3_OD) *δ*: 198.6 (C-4''), 196.3 (C-4), 168.3 (C-7''), 168.1 (C-7), 165.1 (C-8''a), 165.4 (C-5''), 165.2 (C-5), 163.4 (C-8a), 159.0 (C-4'''), 158.6 (C-4'), 130.3 (C-2''', 6'''), 129.1 (C-1'''), 128.8 (C-1'), 128.5 (C-2', 6'), 116.4 (C-3''', 5'''), 116.2 (C- 3', 5'), 105.1 (C-4''a) ,103.9 (C-4a), 97.3 (C-6''), 97.1 (C-6), 96.4 (C-8''), 96.0 (C-8), 83.3 (C-2''), 81.5 (C-2), 50.8 (C-3''), 49.7 (C-3).

Compound II (genkwanol B), a light yellow powder, was positive to FeCl_3_-EtOH reagent. [*α*]_D _= -160° (*c *0.1, MeOH). HR-ESI-MS *m/z*: 557.10805 [M-H] ^- ^.^1^ H-NMR (500 MHz, DMSO-*d*_*6*_) *δ*: 7.09 (2H, d, *J *= 8.5 Hz, H-2''', H-6'''), 6.73 (2H, d, *J *= 8.5 Hz, H-3''', H-5'''), 6.58 (2H, d, *J *= 8.5 Hz, H-2', H-6'), 6.51 (2H, d, *J *= 8.5 Hz, H-3', H-5'), 6.11 (1H, d, *J *= 2.0 Hz, H-8''), 6.04 (1H, d, *J *= 2.0 Hz, H-6''), 5.71 (1H, s, H-6), 4.56 (1H, d, *J *= 8.2 Hz, H-2), 3.55 (1H, m, H-3), 2.59 (1H, dd, *J *= 8.9, 16.7 Hz, H-4), 2.05 (1H, dd, *J *= 5.0, 16.7 Hz, H-4). ^13^C-NMR (125 MHz, DMSO-*d*_*6*_) *δ*: 190.4 (C-4''), 186.3 (C-5), 168.6 (C-7), 167.7 (C-7''), 163.3 (C-5''), 160.1 (C-8''a), 158.1 (C-4'''), 157.9(C-8a), 156.7 (C-4'), 129.8 (C-2''', 6'''), 127.9 (C-1'), 127.3 (C-2', 6'), 122.0 (C-1'''), 114.6 (C-3''', 5'''), 114.4 (C-3', 5'), 109.2 (C-4a) ,100.4 (C-6), 99.8 (C-4''a), 97.0 (C-6''), 96.1 (C-8''), 89.9 (C-2''), 85.0 (C-8), 82.0 (C-2), 79.8 (C-3''), 66.4 (C-3), 27.0 (C-4).

Compound III (genkwanol C), a light yellow powder, was positive to FeCl_3_-EtOH reagent. [*α*]_D _= +15° (*c *0.1, MeOH). HR-ESI-MS *m/z*: 557.10863 [M-H] ^-^. ^1^H-NMR (500 MHz, DMSO-*d *_*6*_) *δ*: 7.07 (2H, d, *J *= 8.5 Hz, H-2''', H-6'''), 6.96 (2H, d, *J *= 8.5 Hz, H-2', H-6'), 6.74 (2H, d, *J *= 8.5 Hz, H-3''', H-5'''), 6.51 (2H, d, *J *= 8.5 Hz, H-3', H-5'), 6.00 (1H, d, *J *= 2.0 Hz, H-8''), 5.93 (1H, d, *J *= 2.0 Hz, H-6''), 5.72 (1H, s, H-6), 4.61 (1H, d, *J *= 6.5 Hz, H-2), 3.77 (1H, m, H-3), 3.16 (1H, d, *J *= 6.5 Hz, H-4), 2.21 (1H, d, *J *= 4.0 Hz, H-4). ^13^C-NMR (125 MHz, DMSO-*d *_*6*_) *δ*: 190.3 (C-4''), 186.3 (C-5), 168.5 (C-7), 167.3 (C-7''), 162.6 (C-5''), 160.2 (C-8''a), 158.1 (C-8a), 157.3 (C-4'''), 156.7 (C-4'), 129.6 (C-2''', 6'''), 127.8 (C-1'), 127.4 (C-2', 6'), 121.9 (C-1'''), 114.6 (C-3''', 5'''), 114.5 (C-3', 5'), 109.5 (C-4a) ,100.48 (C-6), 99.2 (C-4''a), 97.8 (C-6''), 95.7 (C-8''), 90.4 (C-2''), 84.6 (C-8), 81.8 (C-2), 79.9(C-3''), 65.0(C-3), 25.5 (C-4).

Compound IV (stelleranol), a light yellow powder, was positive to FeCl_3_-EtOH reagent. [*α*]_D _= -102° (*c *0.1, MeOH). HR-ESI-MS *m/z*: 557.10913 [M-H] ^-^. CD (*c *6.25×10^-5^, MeOH) Δ*ε *(nm): 0 (394), -6.8 (342), 0 (327), +31.8 (305), 0 (279), -18.6 (259), -7.5 (242), - 21.2 (219), 0 (206). ^1^H-NMR (500 MHz, acetone-*d*_*6*_) *δ*: 7.22 (2H, d, *J *= 8.5 Hz, H-2''', H-6'''), 6.83 (2H, d, *J *= 8.5 Hz, H-3''', H-5'''), 6.71 (2H, d, *J *= 8.5 Hz, H-2', H-6'), 6.62 (2H, d, *J *= 8.5 Hz, H-3', H-5'), 6.16 (1H, d, *J *= 2.0 Hz, H-8''), 6.14 (1H, d, *J *= 2.0 Hz, H-6''), 6.09 (1H, s, H-2''), 5.68 (1H, s, H-6), 4.96 (1H, s, H-2), 4.18 (1H, brs, H-3), 2.65 (1H, d, *J *= 17.5 Hz, H-4), 2.49 (1H, dd, *J *= 3.8, 17.3 Hz, H-4). ^13^C-NMR (125 MHz, acetone-*d*_*6*_) *δ*: 193.1 (C-4''), 188.7 (C-5), 170.4 (C-7), 169.6 (C-7''), 166.1 (C-8''a), 163.1 (C-5''), 160.3 (C-8a), 159.9 (C-4'''), 158.6 (C-4'), 131.7 (C-2''', 6'''), 130.4 (C-1'), 129.2 (C-2', 6'), 124.7 (C-1'''), 116.7 (C-3''', 5'''), 116.5 (C-3', 5'), 111.1 (C-4a) ,102.9 (C-6), 102.0 (C-4''a), 99.0 (C-6''), 98.4 (C-8''), 92.2 (C-2''), 87.5 (C-8), 82.3 (C-2), 82.1 (C-3''), 66.5 (C-3), 28.6 (C-4).

### Cell and virus

Human larynx epidermoid carcinoma cell line (HEp-2, CCL-23) and RSV (long strain, VR-26) were purchased from the American Type Culture Collection (ATCC, USA). The cells were grown in Eagle's minimum essential medium (EMEM) (Gibco, USA) supplemented with 10% fetal bovine serum (FBS) (Gibco, USA), 25 μg/ml gentamicin (Sigma, USA) and 200 mM L-glutamine (Sigma, USA) (growth medium). Virus-infected cells were maintained in EMEM with 1% FBS, 25 μg/ml gentamicin and 200 mM L-glutamine (maintenance medium). All the cells were cultured at 37°C in a humidified atmosphere supplied with 5% CO_2_. Virus titers were determined by the 50% tissue culture infective dose (TCID_50_) method.

### Cytotoxicity assay

Cell viability was tested by the 3-(4,5-dimethylthiazol-2-yl)-2,5-diphenyl tetrazolium bromide (MTT) method as described in previous study [10]. Briefly, 100 μl of two-fold diluted samples were added to a 96-well plate containing confluent cell monolayer in triplicates while the dilution medium without the sample was the control. After 72 hours of incubation, 12 μl of the MTT solution (5 mg/ml in phosphate buffered saline) was added to each well. The trays were further incubated for four hours for the formation of blue formazan. After the supernatant was removed, the blue formazan was solubilized in 100 μl DMSO and the optical density (OD) was measured at 570 nm with a microplate reader.

### Antiviral assay

CPE reduction assay was adopted for screening the *in vitro *antiviral activity as described in the previous study [10]. Briefly, 0.1 ml of 100 TCID_50 _virus suspension and serial two-fold dilutions of the tested samples were added simultaneously to confluent cell monolayers in a 96-well plate. Virus suspension and maintenance medium without samples were added as the virus control and cell control, respectively. The plates were incubated at 37°C in a humidified CO_2 _atmosphere for 3-5 days. The virus-induced CPE was scored against the virus control under a light microscope. Ribavirin (Sigma, USA) was used as positive control in this experiment.

## Results

### Chemical structures of the isolated compounds

**Figure 1:**
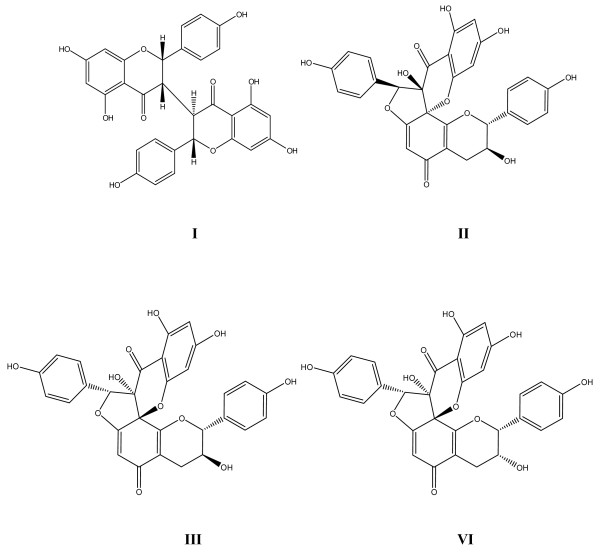
**The chemical structures of biflavonoids I-IV**.

**Figure 2:**
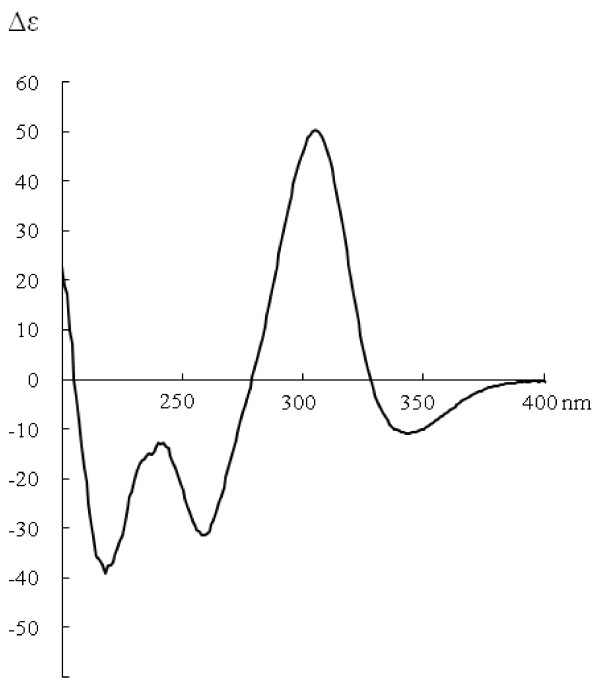
**The CD spectra of biflavonoid IV in MeOH**.

Compound IV was isolated as a light yellow powder. Positive FeCl_3 _reaction showed the presence of phenolic hydroxyl groups. The molecular formula of compound IV was determined to be C_30_H_22_O_11 _by HR-ESI-MS (*m/z *557.10931 [M-H] ^-^, calcd. for C_30_H_21_O_11 _*m/z *557.10893). Proton and carbon signals were assigned by a combination of 1D- and 2D-NMR spectra. The ^1^H-NMR spectrum of compound IV showed signals assignable to a pair of 1,4-disubstituted aromatic rings [*δ *7.22 (2H, d, *J *= 8.5 Hz), 6.71 (2H, d, *J *= 8.5 Hz), 6.83 (2H, d, *J *= 8.5 Hz), and 6.62 (2H, d, *J *= 8.5 Hz)], one 1,2,4,6-tetra substituted aromatic ring [*δ *6.16 (1H, d, *J *= 2.0 Hz), and 6.14 (1H, d, *J *= 2.0 Hz)] and one 3-hydroxy-2,5,6-trisubstituented dihydropyran [*δ *4.96 (1H, s), 4.18 (1H, brs), 2.65 (1H, d, *J *= 17.5 Hz), 2.49 (1H, dd, *J *= 3.8, 17.3 Hz)]. Moreover, two singlet signals were observed at *δ *6.09 (1H, s) and 5.68 (1H, s). In the ^13^C-NMR spectrum of compound IV, two carbonyl carbons (*δ *193.1 and 188.7) and two quaternary carbons with attachment of oxygen atoms (*δ *87.5 and 82.1) were observed in addition to the signals described above. The ^1^H-NMR and ^13^C-NMR data of compound IV were consistent with those of stelleranol [11,12]. There were five chiral carbons including C-2, C-3, C-8, C-2'', and C-3'' in the structure of stelleranol (Figure 1). Among them, the absolute configurations at both C-2 and C-3 were determined to be *R *according to comparison of the NMR data of stelleranol with the published data of (-)-epicatechin [11,12]. The absolute configurations of another three carbons, namely C-8, C-2'' and C-3'', were determined by comparison of the CD spectrum of compound IV with those of genkwanol B (2) and genkwanol C (3) [13]. The CD spectrum of compound IV (Figure 2) was similar to that of genkwanol C, and opposite to that of genkwanol B. Therefore, the absolute configurations at C-2, C-3, C-8, C-2'' and C-3'' positions of stelleranol were fully substantiated to be *R*, *R*, *R*, *R *and *S*.

We also identified compounds I-III to be neochamaejasmin B (I), genkwanol B (II), genkwanol C (III) by comparing their spectra with the published data [13-15]. Compound I was a biflavonone whereas compounds II-IV were stereo isomers of spirobiflavonoids (Figure 1).

### Antiviral activity of the isolated compounds

The *in vitro *antiviral activity of compounds I-IV against RSV was tested with CPE reduction assay and the MTT method. The SI value calculated from the ratio of CC_50 _to IC_50 _was used as an important parameter to evaluate the *in vitro *antiviral activity of the compounds. Compounds II, III and IV showed similar *in vitro *antiviral activity against RSV with IC_50 _values of 9.6, 6.6, 10.2 μM and SI values of 11.0, 21.9, 15.8 respectively whereas compound I did not show anti-RSV effect in its maximal non-cytotoxic concentration (MNCC), the highest concentration tested in the CPE reduction assay (Table 1).

**Table 1:** *In vitro *anti-RSV activity of compounds I-IV (*n *= 3)

Compound	**CC**_**50**_(**μM**) ^**a**^	**IC**_**50 **_(**μM**) ^**b**^	SI
I	29.9 (3.2)	-	-
II	106.1 (5.9)	9.6 (0.7)	11.0
III	145.3 (3.1)	6.6 (1.1)	21.9
IV	161.5 (5.7)	10.2 (0.8)	15.8
Ribavirin	256.1 (3.2)	11.9 (0.2)	21.6

^a ^CC_50 _is the concentration of sample with half maximal inhibition on the growth and survival of HEp-2 cells; data are expressed as mean (SD).

^b ^IC_50 _is the concentration that reduced 50% of CPE in respect to virus control; data are expressed as mean (SD).

-: The sample did not show anti-RSV effect at its NMCC.

## Discussion

Quite a few biflavonoids possess antiviral activities against a number of viruses such as RSV [4,16-20]. Biflavonoids isolated from *Radix Wikstroemiae*, e.g. (+)-nortrachelogenin, genkwanol A, wikstrol B and daphnodorin B, are moderately active against HIV-1 *in vitro *[4]. However, the anti-RSV activity of biflavonoids from *W. indica *has not been discussed before. Three spirobiflavonoids II, III and IV possess similar potent anti-RSV activity to the positive controlled drug ribavirin. In addition, stelleranol is a spirobiflavonoid consisted of five chiral carbons. The absolute configurations of the five chiral carbons in stelleranol are completely substantiated in this study, and the CD spectrum of stelleranol has been given for the first time.

## Conclusion

Neochamaejasmin B, genkwanol B, genkwanol C and stelleranol were isolated from *Radix Wikstroemiae *and the complete absolute configurations of five chiral carbons in stelleranol were substantiated for the first time. Furthermore, the anti-RSV activity of genkwanol B, genkwanol C and stelleranol was reported for the first time.

## Abbreviations

CPE: cytopathic effect; ESI-MS: electrospray ionization-mass spectrometry; HPLC: high performance liquid chromatography; HR-ESI-MS: high resolution-electrospray ionization-mass spectrometry; MTT: 3-(4,5-dimethylthiazol-2-yl)-2,5-diphenyltetrazolium bromide; NMR: nuclear magnetic resonance spectrometry; ^1^H-MNR: proton magnetic resonance spectrometry; ^13^C-NMR: carbon magnetic resonance spectrometry; RSV: respiratory syncytial virus; SI: selective index; TLC: thin layer chromatography; UV: ultraviolet; HIV: human immunodeficiency virus.

## Competing interests

The authors declare that they have no competing interests.

## Authors' contributions

WH performed the chemical isolation, antiviral tests and manuscript preparation. XZ assisted in chemical isolation and manuscript preparation. YW and HYC conducted the antiviral experiments. VECO designed the antiviral experiments and revised the manuscript. YL designed the study and revised the manuscript. WY designed the overall study. All authors read and approved the final version of the manuscript.
